# Methicillin-resistant *Staphylococcus aureus* in Taiwan

**DOI:** 10.3201/eid1111.050367

**Published:** 2005-11

**Authors:** Feng-Jui Chen, Tsai-Ling Lauderdale, I-Wen Huang, Hsiu-Jung Lo, Jui-Fen Lai, Hui-Yin Wang, Yih-Ru Shiau, Pei-Chen Chen, Teruyo Ito, Keichii Hiramatsu

**Affiliations:** * National Health Research Institutes, Zhunan, Taiwan; † Juntendo University, Tokyo, Japan

**Keywords:** Methicillin-resistant Staphylococcus aureus, MRSA, Community-associated MRSA, Panton-Valentine leukocidin (PVL), SCC*mec* V, dispatch

## Abstract

We found a virulent closely related clone (Panton-Valentine leukocidin–positive, SCC*mec* V:ST59) of methicillin-resistant *Staphylococcus aureus* in inpatients and outpatients in Taiwan. The isolates were found mostly in wounds but were also detected in blood, ear, respiratory, and other specimens; all were susceptible to ciprofloxacin, gentamicin, and trimethoprim-sulfamethoxazole.

Although most methicillin-resistant *Staphylococcus aureus* (MRSA) illness and death are associated with healthcare facilities (H-MRSA), isolates from community-associated MRSA (C-MRSA) infections have been obtained with increasing frequency in the last few years in different countries, including Taiwan (*1**–**5*). The changing epidemiology of MRSA has become an important public health concern worldwide (*1**,**4*). MRSA arises when *S. aureus* organisms acquire a large mobile genetic element called staphylococcal cassette chromosome *mec* (SCC*mec*) (*6*). Most H-MRSA strains possess either SCC*mec* II or III; most C-MRSA strains possess SCC*mec* IV (*7**–**9*). Recently, a novel type V SCC*mec* type was characterized and found in a C-MRSA isolate from Australia (*10*). With the exception of variable resistance to erythromycin, C-MRSA strains are generally susceptible to other non-β-lactam antimicrobial agents, in contrast to most H-MRSA, which are typically resistant to many of the non-β-lactam agents (*1**,**4**,**9*). Another characteristic of C-MRSA is the production of Panton-Valentine leukocidin (PVL), an extracelluar cytotoxin involved in primary skin infections and pneumonia (*2**–**4*).

We conducted a study to characterize the molecular epidemiology of selected MRSA isolates from the Taiwan Surveillance of Antimicrobial Resistance (TSAR), a national surveillance program of inpatient and outpatient clinical isolates in Taiwan (*11*). We describe the finding of a virulent closely related clone of MRSA and its prevalence in Taiwan.

## The Study

A total of 398 and 865 nonduplicate *S. aureus* isolates were collected from March to May 2000 from 21 hospitals and from July to September 2002 from 26 hospitals, respectively, as part of the TSAR collection (*11*). The proportions of isolates for the 2 years were similar for outpatients (27.5% in 2000 and 28.9% in 2002); the rest of the isolates were from inpatients. The most common specimen type was wound, which accounted for 35.4% and 49.2% of all *S. aureus* isolates in 2000 and 2002, respectively. Antimicrobial susceptibility was determined on the basis of results of MICs obtained from a broth microdilution method, following the guidelines of the National Committee for Clinical Laboratory Standards (*12*) by using custom-designed Sensititre plates (Trek Diagnostics, East Essex, United Kingdom). Overall, MRSA accounted for 238 (59.8%) of 398 *S. aureus* isolates in 2000 and 475 (54.9%) of 865 *S. aureus* isolates in 2002.

**Figure A1:**
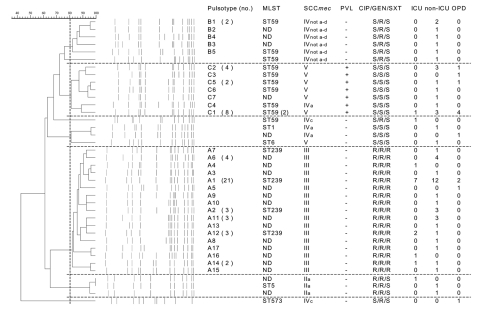
Molecular characterization (pulsotype, MLST, SCCmec, PVL), antimicrobial (CIP/GEN/SXT) susceptibility profile, and patient source of 80 methicillin-resistant Staphylococcus aureus isolates from 4 hospitals in Taiwan. CIP, ciprofloxacin; GEN, gentamicin; SXT, trimethoprim/sulfamethoxazole; R, resistant; S, susceptible; pulsotype, pulsed-field gel electrophoresis type; MLST, multilocus sequence typing result in sequence type (ST); SCCmec, staphylococcal cassette chromosome mec type; PVL, Panton-Valentine leukocidin; (no.) following pulsotype and MLST results indicates the number of isolates if >1; numbers below ICU, non-ICU, and OPD indicate number of patients from intensive care unit, nonintensive care inpatient, and outpatients, respectively.

To obtain an overall understanding of MRSA throughout Taiwan, we first chose 80 MRSA isolates (68 inpatient and 12 outpatient isolates) collected in 2002 from 4 hospitals located in the north, middle, south, and east regions of Taiwan. Pulsed-field gel electrophoresis was performed according to a published protocol (*9*), and pulsotypes were assigned to clusters of isolates with >80% similarity from the dendrograms. SCC*mec* typing and PVL gene detection were performed according to published protocols (*7**,**8**,**10*). Multilocus sequence typing (MLST) was performed on randomly selected strains from major pulsotypes, and the sequence type (ST) was assigned by using the MLST database (http://www.mlst.net) (*13*). Three major clusters (pulsotypes) were found, including 47 (58.8%) pulsotype A, 7 (8.8%) pulsotype B, and 18 (22.5%) pulsotype C (Figure A1). All 47 pulsotype A isolates had SCC*mec* III; 4 isolates tested by MLST had ST239. In addition to being resistant to clindamycin (94%), erythromycin (100%), and tetracycline (100%), all pulsotype A isolates were resistant to ciprofloxacin (CIP), gentamicin (GEN), and trimethoprim/sulfamethoxazole (SXT). The 7 pulsotype B isolates possessed SCC*mec* IV but were not of the 4 known IV subtypes (IV not a–d); all were CIP- and SXT-susceptible but GEN-resistant. Seventeen of the 18 isolates in pulsotype C possessed SCC*mec* V; the other had SCC*mec* IVa; all 18 were CIP/GEN/SXT-susceptible. Of the 10 isolates tested by MLST from pulsotype B (3 isolates) and pulsotype C (7 isolates), all had ST59. Only pulsotype C isolates were PVL-positive.

Because we found a large percentage (21.3%, 17/80) of SCC*mec* V:ST59, PVL-positive clones, we selected an additional 69 CIP/GEN/SXT-susceptible MRSA isolates (25 from 2000, 44 from 2002) to determine what portion of them had the same clone. These isolates were from intensive care unit (ICU) and non-ICU inpatients, plus outpatients from 15 hospitals in 2000 and 21 hospitals in 2002 (excluding the 4 hospitals already tested). Fifteen (60%) of the 25 isolates from 2000 and 32 (72.7%) of the 44 isolates from 2002 belonged to pulsotype C, had SCC*mec* V, and were PVL-positive. Of the 4 isolates characterized by MLST, 2 were ST59, 1 was ST388, and 1 was a new ST; the last 2 isolates differed from ST59 by 1 nt each in *gmk* and *arcC* genes, respectively.

**Figure A2:**
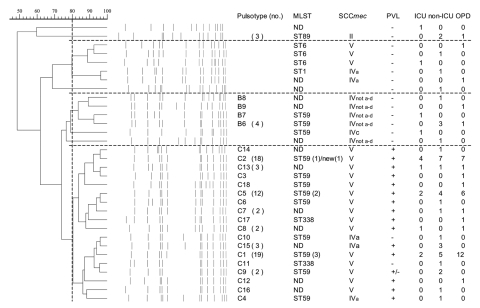
Molecular characterization (pulsotype, MLST, SCCmec, PVL) of 90 ciprofloxacin/gentamicin/SXT-susceptible methicillin-resistant Staphylococcus aureus isolates from Taiwan hospitals in 2000 and 2002. Pulsotype, pulsed-field gel electrophoresis type; MLST, multilocus sequence typing (MLST) result in sequence type (ST); SCCmec, staphylococcal cassette chromosome mec type; PVL, Panton-Valentine leukocidin; (No.) following pulsotype and MLST results indicates the number of isolates if >1; numbers below ICU, non-ICU, and OPD indicate number of patients from intensive care unit, nonintensive care inpatients, and outpatients, respectively.

When the 2 groups of isolates were combined, a total of 90 CIP/GEN/SXT-susceptible MRSA were studied (Figure A2). These isolates were mostly resistant to clindamycin (89%), erythromycin (92%), and tetracycline (82%). Of these 90 isolates, 68 (75.6%) were PVL-positive and belonged to same pulsotype C; 64 (71.1%) had SCC*mec* V. MLST performed on 12 isolates found 10 to be ST59; the other 2 (ST338 and the new ST) are closely related to ST59. Forty-eight of these 64 isolates were from wounds (75%), but isolates were also found in respiratory (6 isolates), ear (3 isolates), blood (2 isolates), urine (2 isolates), and catheter and other body site (3 isolates) specimens. Only half (32 isolates) were from outpatients; the rest were from ICU (10 isolates) and non-ICU (22 isolates) inpatients. Two ICU isolates were from hospital-acquired infections. Because 189 (26.5%) of the 713 MRSA isolates from the 2000 and 2002 collections were CIP/GEN/SXT-susceptible, and because 64 (71.1%) of the 90 CIP/GEN/SXT-susceptible MRSA we studied were PVL-positive, SCC*mec* V:ST59, and belonged to pulsotype C, an estimated 18.8% (26.5% × 0.711 = 18.8%) of MRSA isolates in Taiwan could be this closely related virulent clone.

The reason for the high prevalence of this virulent clone (pulsotype C:ST59, SCC*mec* V, PVL-positive) of MRSA in Taiwan in both inpatients and outpatients is not known. Data on the prevalence of SCC*mec* V are still limited, and ST59 has been described infrequently. A recent longitudinal study of MRSA isolates in the San Francisco area found that the ST59-SCC*mec* IV has increased steadily from 1999 to become 1 of the 4 major clones associated with C-MRSA (*14*).

Production of the PVL cytotoxin is considered a genetic marker for C-MRSA, and although PVL-positive MRSA have usually been associated with skin and soft tissue infections, severe and fatal infections, such as necrotizing pneumonia, have been reported (*2**–**4*). Thus, PVL may confer an additional virulence advantage for this particular clone of MRSA in Taiwan. Other possible explanations are that the less resistant C-MRSA can grow faster than multidrug-resistant H-MRSA and that SCC*mec* IV and V carried by C-MRSA may have the advantage over SCC*mec* I–III carried by H-MRSA because they are smaller and more transferable; both of these putative advantages may contribute to their propagation (*10**,**15*).

The close relatedness and high prevalence of this virulent pathogen argue for a clonal expansion advantage of this particular clone. Outbreaks of C-MRSA infections caused by SCC*mec* IV (IVa) have been reported in several countries (*4*). Since our genotyping results showed that MRSA isolates possessing SCC*mec* V and PVL in Taiwan are clonally related, we cannot rule out the possibility of outbreaks due to this particular clone in some areas. However, our isolates came from multiple hospitals throughout the 4 geographic regions of Taiwan. Our data also showed that this particular clone was already present in 2000. In addition, this particular clone was found not only in outpatients but also in ICU and non-ICU inpatients, including in hospital-acquired infections. These findings indicate that this clone has migrated into the hospital environment; moreover, it can cause more severe infections, as shown by its presence in blood, respiratory, ear, and other specimens.

## Conclusions

Our analysis of MRSA isolates collected in 2000 and 2002 indicated that a virulent clone of MRSA (pulsotype C:ST59, SCC*mec* V and PVL-positive), which caused wound infections primarily but also other potentially more serious infections, is highly prevalent in Taiwan inpatient and outpatient settings. Recognition of this clone can be facilitated by its antimicrobial susceptibility profile. Because the resistance pattern of these isolates differs from that of traditional H-MRSA strains, the antimicrobial susceptibility profile has important implications for treatment. Understanding the roles these strains play in MRSA epidemiology helps physicians choose the most appropriate treatment. Prompt and judicious management and infection control measures should help deter further spread of this virulent pathogen.
